# A Rare and Misdiagnosed Entity Paroxysmal Nocturnal Hemoglobinuria: A Case Report

**DOI:** 10.7759/cureus.14902

**Published:** 2021-05-08

**Authors:** Biraj Pokhrel, Sandesh Gautam, Shambhu Khanal, Nishan B Pokhrel, Anjan Shrestha

**Affiliations:** 1 Internal Medicine, Tribhuvan University, Institute of Medicine, Maharajgunj Medical Campus, Kathmandu, NPL

**Keywords:** anemia, erythrocytes, hemoglobinuria, hemolysis, thrombosis

## Abstract

Paroxysmal nocturnal hemoglobinuria (PNH) is a rare, acquired stem cell disorder manifesting as non-immunological hemolytic anemia, hemoglobinuria, unusual thrombosis, and renal impairment due to deficiency of glycosylphosphatidylinositol (GPI) linked proteins in red blood cells. Patients present with features of chronic non-immune intravascular hemolysis, unexplained anemia, and thrombosis at unusual sites. It is often misdiagnosed and treated as anemia due to a low degree of suspicion. In resource-limited settings, the low degree of suspicion and paucity of investigations are the major diagnostic challenges. The even bigger challenge remains in the affordability of definitive treatment after a diagnosis has been made. Herein, we present a case of PNH in a 26-year man from rural Nepal who went undetected during the initial presentation of hemolytic anemia and later presented to us with hemolytic anemia and gastrointestinal symptoms. We made the provisional diagnosis based on the clinical presentations. However, we faced challenges in reaching the final diagnosis and providing the definitive treatment due to financial constraints and limited resources. Any patient presenting with features of chronic non-immune intravascular hemolysis, unexplained anemia, and unusual thrombosis should prompt the consideration of PNH.

## Introduction

Paroxysmal nocturnal hemoglobinuria (PNH) is a rare hematological disease resulting from an acquired somatic mutation in a multipotent hematopoietic stem cell (HSC). The mutation results in the production of blood cells that are deficient in the glycosylphosphatidylinositol (GPI) linked surface proteins [1,2]. The acquired mutation occurs in the PIGA gene (phosphatidylinositol glycan anchor biosynthesis, class A) responsible for the synthesis of GPI anchor protein [2,3]. This PIGA gene is located in the short arm of the X chromosome and a single “hit” (i.e., a mutation in a single allele of the gene) to this gene can produce phenotype in both males and females (as males have a single X chromosome and females have only single active X chromosome due to lyonization) [3]. GPI is a glycolipid that anchors proteins like blood group antigens, adhesion molecules, and complement regulatory proteins (CD55, CD59, and C8 binding protein) to the cell membrane.

Complement regulatory proteins protect blood cells (RBCs, white blood cells, and platelets) against complement-mediated damage. CD55 inhibits C3 convertases and CD59 blocks the formation of the membrane attack complex (MAC) [4]. Affected PNH RBCs, which lack surface complement regulatory proteins, are highly susceptible to injury by unopposed action of terminal complement and subsequent chronic non-immune intravascular hemolysis, which is the primary clinical manifestation of the disease. Other manifestations include smooth muscle dystonias, unexplained thrombosis at unusual sites like the hepatic vein (Budd-Chiari syndrome) and bone marrow failure [5,6]. Being rare, it is often misdiagnosed due to which a significant time is lost on reaching the definitive diagnosis [1]. Treatment with eculizumab, a humanized monoclonal antibody against complement protein C5, and allogeneic bone marrow transplantation (BMT) are the only widely proven effective therapies for patients with PNH [3].

To the best of our knowledge, only a single case of PNH has been reported from Nepal in a young male [7]. It is probable that too many cases are being misdiagnosed due to financial constraints and limited resources in our setting. Herein, we report the case of a 26-year-old man from rural Nepal, who was inappropriately treated for four years prior to the presentation. The strong clinical suspicion guided us through a series of investigations, which confirmed the diagnosis of PNH.

## Case presentation

A 26-year-old male presented to our emergency with upper abdominal pain and multiple episodes of vomiting for 12 days, preceded by fatigue and yellowish discoloration of both eyes for two months. He had an insidious onset of progressive fatigue and weakness for two months, which was affecting his daily activities. He also had gradually progressive yellowish discoloration of the eyes and dark-colored urine, especially in the morning. A constant, dull aching, non-radiating abdominal pain was prominent on the right upper quadrant, was exacerbated by food, and relieved after analgesics and pantoprazole. He also reported multiple episodes of vomiting, which was not mixed with bile or blood. He had no history of itching, clay-colored stool, or fever.

The patient had similar symptoms four years back for which he was considered to be anemic and received eight pints of whole blood in a nearby hospital. However, after receiving symptomatic treatment, he was discharged. He was not worked up further. He had no other significant past medical or surgical history. He consumed about six units of homemade alcohol per day for the last five years. There was no family history of similar illnesses.

On examination, his blood pressure was low. He appeared dehydrated, pale, and icteric. Systemic examination did not reveal significant findings. Fluid resuscitation was immediately started and baseline investigations were done.

Investigations showed anemia, deranged liver and renal functions (Table 1).

**Table 1:** Laboratory data

Parameters	At the time of admission	Normal reference used
1. Haemoglobin	10.2 gm%	12-18 gm%
2. Packed cell volume	10.2%	36%-54%
3. RBCs	3.24 million/cubic mm	4.5-5.5 million/cubic mm
4. Mean red cell volume	29.8%	76%-98%
5. Reticulocyte count	2%	0.5-%1.5%
6. Renal function test		
a. Creatinine	146 µMol/L	60-115 µMol/L
b. Urea	10.6 mMol/L	1.6-7.0 mMol/L
c. Na^+^	136 mEq/L	135-146 mEq/L
d, K^+^	2.0 mEq/L	3.5-5.2 mEq/L
7. Liver function test		
a. Total bilirubin	104 µMol/L	3-21 µMol/L
b. Direct bilirubin	22 µMol/L	0-4 µMol/L
c. Alanine aminotransferase	32 U/L	5-45 U/L
d. Aspartate aminotransferase	126 U/L	5-40 U/L
e. Total protein	8.2 g/L	60-80 g/L
f. Albumin	4.8 g/dL	3.5-5.5 g/dL
8. Lactate dehydrogenase	1,854 U/L	<460 U/L

Serological spot tests for human immunodeficiency virus (HIV) antibody, hepatitis B surface antigen (HBsAg), and hepatitis C virus (HCV) antibody were non-reactive. Routine and microscopic examination of urine was normal. With the clinical picture of hemolytic anemia with pre-renal azotemia, he was admitted for further evaluation.

Peripheral blood smear revealed normocytic, normochromic RBCs. Serum lactate dehydrogenase was raised (1,854 U/L, normal range <460). The stool examination was negative for parasites. Iron profile (serum iron, total iron-binding capacity, ferritin) was within normal limits.

On the ground of normal peripheral blood smear, raised lactate dehydrogenase level, and unconjugated hyperbilirubinemia, we performed direct Coomb’s test to rule out immune-mediated destruction, which came out to be negative. The test for urine hemosiderin was positive. The glucose-6-phosphate dehydrogenase test also showed normal enzyme levels.

Ultrasonography (USG) of the abdomen revealed cavernoma formation from the main portal vein and its intrahepatic branches, pointing towards the possibility of extrahepatic portal vein thrombosis. Esophagogastroduodenoscopy showed evidence of bile reflux pangastritis without varices. We considered PNH at this point and proceeded to confirm the diagnosis.

**Figure 1:**
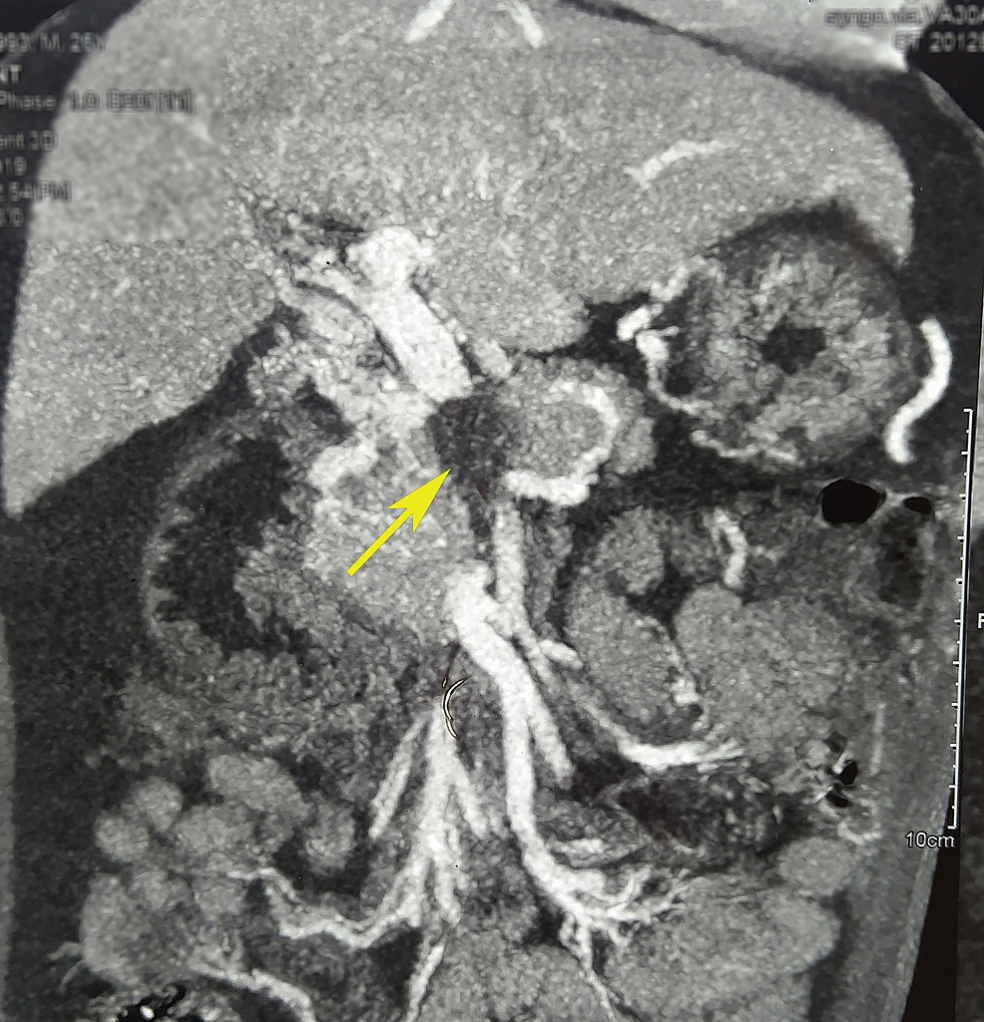
Coronal view of contrast CT (venous phase) showing thrombus in the main portal vein and the distal superior mesenteric vein (yellow arrow). CT, computed tomography

**Figure 2:**
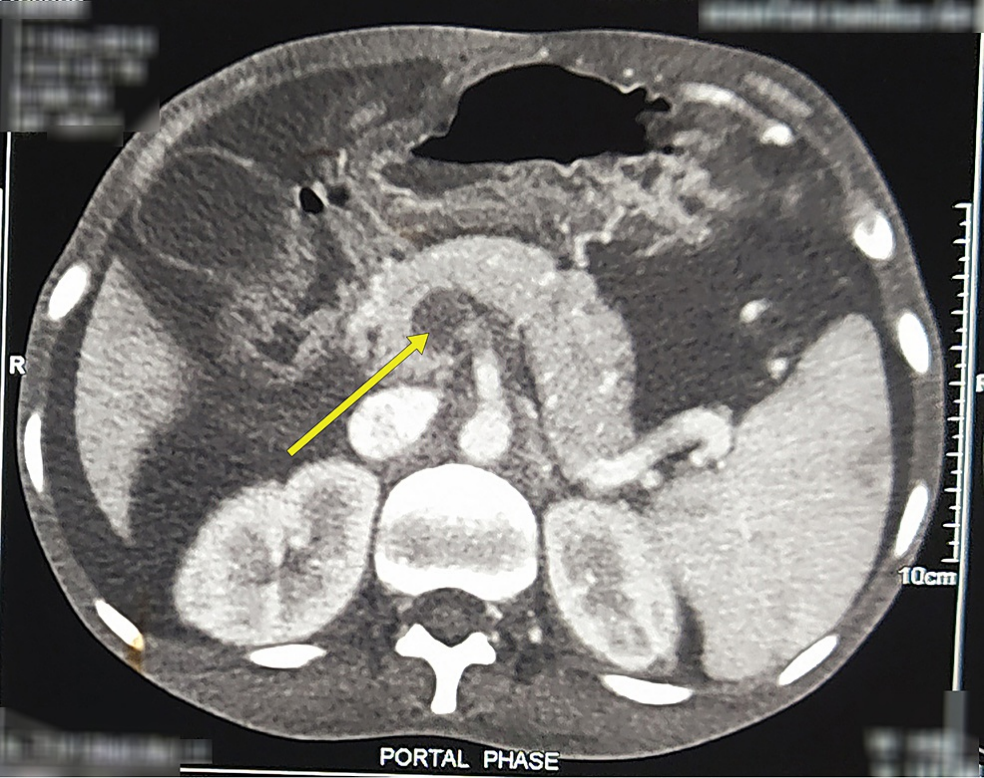
Axial view of contrast CT (portal phase) showing occluding thrombus in the splenic vein and the portal vein (yellow arrow). CT, computed tomography

Because of the financial constraints, we did the Hams test initially, which was positive. Computed tomography arterial portography (CTAP) was possible only after a week when he got donations. It revealed partial occluding thrombus in the main portal vein and complete occluding thrombus in distal superior mesenteric vein and splenic vein with cavernoma formation in the porta hepatis, peripancreatic and perigastric regions (Figures 1, 2).

Finally, PNH flow cytometry was done, which showed PNH clones within RBCs (38.5%), granulocytes (97.99%), and monocytes (97.40%) (Table 2).

**Table 2:** Flow cytometry analysis of PNH FLAER, fluorescein-labeled proaerolysin PNH, Paroxysmal nocturnal hemoglobinuria

	Results	Reference
Red blood cells (Gating marker: CD235a)		
Type II (Partial CD59 deficiency)	11.70 %	<1
Type III (Complete CD59 deficiency)	26.80%	<1
Type II & Type III (Combined deficiency)	38.50%	<1
White Blood Cells - Monocytes (Gating marker: CD33/CD64)		
FLAER, CD14, and CD55 deficiency	97.40%	<1
White blood cells - Granulocytes (Gating marker: CD15)		
FLAER and CD24/CD55 deficiency	97.99%	<1

The treatment after the diagnosis was even more challenging. Eculizumab, the only effective treatment, could not be afforded by our patient due to low socio-economic conditions. Additionally, he was from the rural area of Nepal from where it takes around two days to reach our center. We offered him folic acid, anticoagulation, and a steroid (in a tapering fashion). Adequate anticoagulation was achieved with warfarin tablets bridged with low molecular weight heparin.

## Discussion

PNH is a rare and fatal hematological disease with diagnostic challenges in resource-limited settings. With many of the cases being likely to be undiagnosed, the reported incidence is about one to 10 cases per million population [1]. Patients may present with the features of hemolytic anemia and unexplained thrombosis as in classical PNH or may present in the setting of bone marrow disorder (aplastic anemia). However, there may be no presentation in some cases [8].

The clinical spectrum of PNH differs broadly based on the size of PNH clones. Patients having large PNH clone sizes (as measured in abnormal granulocytes in percentage) often have increased risk for thrombosis (greater than 61%) as well as hemoglobinuria, abdominal pain, oesophageal spasm, and impotence, whereas those with relatively small clone sizes ( less than 10%) have only mild or subclinical hemolysis [8,9]. Thrombosis, the rare and the most serious complication affecting the quality of life, has mortality up to 40% in the untreated patient [6,10]. In our case, a large proportion (97.99%) of PNH granulocyte clones were present, which justified the thrombosis. The presenting symptoms in our case were fatigue and upper abdominal pain, as are the common presentations in PNH [5]. Other manifestations are back pain, oesophageal spasm, dysphagia (difficulty swallowing), and erectile dysfunction. These symptoms are due to the direct consequences of free hemoglobin. Free hemoglobin scavenges the free nitric oxide (NO), which maintains the smooth muscle cell relaxation. Fatigue and upper abdominal pain are vague symptoms, which might be confused with other diseases like acute gastritis. Probably, our patient had been prescribed pantoprazole for the same reason. Similarly, our patient had multiple blood transfusions for anemia without diagnostic consideration of PNH.

Due to the rarity of this entity, it is misdiagnosed often with hematuria, iron deficiency, hemolytic, megaloblastic or refractory anemia, and myelodysplastic syndromes [11]. As an example, Yin et al. reported a case of PNH which was misdiagnosed and treated as iron deficiency anemia for three years [12]. Similarly, Paudyal et al. from Nepal reported a case that was misinterpreted as megaloblastic anemia initially [7]. Due to the presence of fatigue, it may be misdiagnosed and treated as anemia for years, as was with our case. Later, clinical picture, features of non-immune mediated chronic intravascular hemolysis, and evidence of unexplained thrombosis at unusual sites from CTPA raised our suspicion towards PNH.

Though not so specific, we did Hams test because of financial constraints [13,14]. This test cannot detect the low level of PNH cells and requires the presence of at least 10% hemolysis for the test to be positive [15]. The test was positive in our case. So, the flow cytometry was performed to gain better confidence in diagnosing it with high sensitivity and specificity [16]. This showed a large granulocyte clone (greater than 50%) relating to the thrombotic and the hemolytic picture of the patient.

Eculizumab, a humanized monoclonal antibody against the terminal complement protein C5, has been shown to improve the quality of life for patients with hemolytic PNH. It reduces hemolysis, thrombosis, and transfusion requirements [17]. Complement C5 is split by C5 convertase into C5a and C5b, which would ultimately lead to the formation of MAC. However, this MAC binds and permeabilizes bacterial walls, thereby killing the microorganism. So, there is an increased chance of infections using eculizumab. Multicenter phase 3 study done by Brodsky et al. showed pyrexia, headache, abdominal distension, viral infection, anxiety, and renal impairment to be the serious adverse events related to the drug [17]. BMT, the only curative therapy for PNH, is associated with significant morbidity and mortality [3,18].

Some authors also mention the use of brief pulses of corticosteroids as a treatment option in attenuating acute hemolytic exacerbation. But it is a matter of debate without any experimental data to support the importance of corticosteroids in ameliorating acute exacerbations. It may reduce the severity and duration of crisis avoiding the risk of untoward events linked with its long-term use [8,19]. In our case, corticosteroids decreased acute exacerbations. Nevertheless, its side effect on long-term use cannot be avoided.

## Conclusions

PNH is a rare clonal disorder characterized by hemolytic anemia, bone marrow failure, and thrombosis at unusual sites. Often it is misdiagnosed and treated as anemia due to a low degree of suspicion. In resource-limited settings, the low degree of suspicion and paucity of investigations are the major diagnostic challenges. The even bigger challenge remains in the affordability of definitive treatment after a diagnosis has been made. Any patients presenting with features of chronic non-immune intravascular hemolysis, unexplained anemia, and unusual thrombosis should prompt the consideration of PNH.
